# Genomic-enabled prediction models using multi-environment trials to estimate the effect of genotype × environment interaction on prediction accuracy in chickpea

**DOI:** 10.1038/s41598-018-30027-2

**Published:** 2018-08-03

**Authors:** Manish Roorkiwal, Diego Jarquin, Muneendra K. Singh, Pooran M. Gaur, Chellapilla Bharadwaj, Abhishek Rathore, Reka Howard, Samineni Srinivasan, Ankit Jain, Vanika Garg, Sandip Kale, Annapurna Chitikineni, Shailesh Tripathi, Elizabeth Jones, Kelly R. Robbins, Jose Crossa, Rajeev K. Varshney

**Affiliations:** 1 International Crops Research Institute for the Semi-Arid Tropics (ICRISAT), Hyderabad, India; 2 University of Nebraska-Lincoln, Lincoln, NE 68583 USA; 3 Indian Agricultural Research Institute (IARI), Delhi, India; 4 IPK-Gatersleben, D-06466 Gatersleben, Germany; 5 Cornell University, Ithaca, NY 14850 USA; 6 International Maize and Wheat Improvement Center (CIMMYT), Mexico, Mexico

## Abstract

Genomic selection (GS) by selecting lines prior to field phenotyping using genotyping data has the potential to enhance the rate of genetic gains. Genotype × environment (G × E) interaction inclusion in GS models can improve prediction accuracy hence aid in selection of lines across target environments. Phenotypic data on 320 chickpea breeding lines for eight traits for three seasons at two locations were recorded. These lines were genotyped using DArTseq (1.6 K SNPs) and Genotyping-by-Sequencing (GBS; 89 K SNPs). Thirteen models were fitted including main effects of environment and lines, markers, and/or naïve and informed interactions to estimate prediction accuracies. Three cross-validation schemes mimicking real scenarios that breeders might encounter in the fields were considered to assess prediction accuracy of the models (CV2: incomplete field trials or sparse testing; CV1: newly developed lines; and CV0: untested environments). Maximum prediction accuracies for different traits and different models were observed with CV2. DArTseq performed better than GBS and the combined genotyping set (DArTseq and GBS) regardless of the cross validation scheme with most of the main effect marker and interaction models. Improvement of GS models and application of various genotyping platforms are key factors for obtaining accurate and precise prediction accuracies, leading to more precise selection of candidates.

## Introduction

Chickpea (*Cicer arietinum* L.) is the second most important food legume crop with genome size of ~740 Mb^1^. It’s high protein content and nutritional value make it important for human consumption as well as animal feed^2^. Moreover, chickpea has an important role in vegetarian diet because it is high in dietary fiber, folate, iron and phosphorus content^3^. Chickpea is mostly grown in the arid and semi-arid regions, predominantly in developing countries (more than 70% of its cultivated area) and is a major source of livelihood for resource poor farmers living in South Asia and Sub-Saharan Africa^4^. Chickpea suits very well in crop rotation programs as it has the capacity to fix soil N_2_ using symbiotic nitrogen fixation process.

Various biotic (*Ascochyta* blight, *Fusarium* wilt, *Helicoverpa* pod borer, *Botrytis* grey mold) and abiotic (heat, cold and drought) factors adversely affect chickpea yields globally^5,6^. Global climatic changes including erratic rainfall, are leading to drought of various intensities in most of the chickpea growing regions, thereby severely affecting the chickpea production. Considering the impact of these stresses on yield, it is very important to develop improved varieties that not only sustain but result in enhanced chickpea production under these adverse conditions.

As per one of the estimates, food production needs to go up by 70%, with an extreme production pressure on developing countries to double their produce to achieve the nutritional security for an estimated world population of 9.1 billion by 2050 (FAO). In order to cope with an elevated food demand and declining productivity of the crops, breeding efforts combined with genomic approaches popularly known as genomics-assisted breeding (GAB)^7^, holds the potential to enhance the rate of genetic gains. Until few years back, chickpea was considered as an orphan crop due to the scarcity of genomic resources, therefore not much effort to deploy GAB for chickpea improvement could be initiated. However, recent advances in next generation sequencing (NGS) technology has brought down the genotyping cost significantly enabling the generation of huge amounts of genomic resources in much less time and with significantly decreased cost. Using NGS technology, the draft genome of chickpea was completed, and a large number of marker resources were made available^1^. In addition to the draft genome, several large scale re-sequencing efforts using NGS based whole genome re-sequencing have generated millions of markers that can be deployed in GAB for chickpea improvements^8,9^. This vast amount of information enabled the researchers and breeders to design improved strategies for development of improved chickpea varieties. The current average chickpea productivity is less than 1 t/ha, and GAB approaches hold the potential to increase this significantly^10^. Improved chickpea lines with higher yield under rainfed conditions have been developed using marker assisted backcrossing (MABC) in the JG 11 background (a leading desi type chickpea variety widely grown in India) by introgressing the “*QTL-hotspot”* genomic region from the donor parent ICC 4958^11^. Similarly using MABC, improved chickpea lines with enhanced resistance to *Fusarium wilt* and *Ascochyta* blight were developed by introgressing the *foc1* locus and two quantitative trait loci (*QTLs*) *viz. ABQTL-I* and *ABQTL-II*, respectively, in the genetic background of C 214 (another elite chickpea cultivar)^12^. Inspired by the success of these improved lines, several efforts are underway to develop improved chickpea varieties using MABC.

Genomic selection (GS) is becoming a popular technique enabling breeders to select lines using genome-wide marker data before estimating their actual performance in the fields. GS eliminates multiple rounds of phenotypic selection using marker data and thereby contributes to enhanced rate of annual genetic gain per unit of time and cost^13^. In GS, individuals with genotypic and phenotypic information are used to model relationships between phenotype and genotype of observed lines, and then the model enables the predictions of phenotypes for unobserved lines using their marker profile. GS uses the genome-wide marker profile for estimating the performance of lines based on the genomic estimated breeding value (GEBV) offering superiority to marker assisted selection (MAS)^14^ where only markers that are above a specific significant threshold are included in the model. Various parametric and nonparametric approaches among different statistical methods have been explored to develop GS models^15–20^. In addition, several studies comparing simulated and empirical data have been conducted^21–23^.

GS has been successfully used in breeding programs contributing to improved yield and other agronomically important traits for different crops^24–26^. However, the presence of genotype × environment (G × E) interactions complicates the selection of stable lines, negatively affecting the heritability of the traits and response to selection. It is expressed as a change in ranks of the performance of a set of lines from one environmental condition to another. Hence accounting and modeling for G × E interaction in genomic prediction models could help breeders to select lines with optimal overall performance across environments and in specific target environments as well.

The productivity and the nitrogen content of chickpea has been found to be affected by environmental factors such as nitrogen nutrition, phosphorus content, drought stress, and pathogens^27,28^. Adapting GS techniques to model the G × E interaction can help enhance chickpea production. Recently, a few GS models have been developed allowing the incorporation of the G × E interaction^29,30^. While Burgueño *et al*.^29^ accounts for the G × E using structured co-variances to model relationships among environments, Jarquin *et al*.^30^ allows the inclusion of environmental information (e.g., temperature, nitrogen level, soil moisture, etc.) to model these relationships via covariance structures. The model described by Jarquin *et al*.^30^ is also known as the multiplicative reaction norm model (MRNM). The reaction norm model for assessing G × E interaction has been widely used in recent years as it decomposes the total phenotypic variance into genotype, environments, and G × E components that are used in the various prediction models. Jarquin *et al*.^30^ has shown the use of the models for assessing prediction accuracy with genomic main effects and G × E interaction and demonstrated that including interaction into the model substantially increase prediction accuracy in wheat trails including sets of environmental covariables.

The current study deals with the incorporation of the G × E interaction into the GS model to enable precise selection of lines in different environments, with the objective of evaluating GS models for predicting phenotypes using marker information in chickpea by means of the reaction norm model of Jarquin *et al*.^30^. We utilized a set of models including an alternative version of the MRNM which do not require the environmental information but the identification number of the tested environments has to be specified. We evaluated the accuracy of predictions in a trial basis for different site-by-year-management combinations. The main objectives were to compare genomic-enabled prediction accuracy of thirteen different GS models for eight traits and three cross validation (CV) scenarios mimicking prediction problems that breeders might face in fields (sparse testing prediction, CV2; prediction of newly developed lines, CV1; prediction of environments that were never tested, CV0). Predictions were estimated using two different sequencing platforms (DArTseq and Genotyping by Sequencing (GBS)) individually, as well as combined.

## Results

### Genotyping data

The approach DArTseq resulted in 1,568 SNPs, and the GBS resulted in 88,845 SNPs. As described by Roorkiwal *et al*.^10^ the estimated polymorphism information content (PIC) for DArTseq varied from 0.01 to 0.38 across the genotypes with a mean PIC value of 0.19. However, high throughput sequencing (GBS) on HiSeq 2500 platform resulted in 196 million reads producing 721,860 total tags with a minimum tag count of 10 and alignment rate of 83.89%. Further, filtered sequencing reads were analyzed for SNP identification using the TASSEL-GBS pipeline. As a result, 88,845 SNPs were identified with the maximum number of SNPs on CaLG04 (15,146, 17.05%) and minimum on CaLG08 (5,379, 6.05%). The estimated PIC for GBS SNP varied from 0.01 to 0.5 across the genotypes with a mean PIC value of 0.3.

### Comparison of performance of different GS models across different traits

Performance of each model varied across the eight traits and the different random cross-validations schemes (CV0, CV1 and CV2, thus none of the models was found clearly superior to another. However, focusing on only one trait at a time some interesting patterns could be identified.

In terms of assessing the prediction accuracy of a model based on the correlation between the observed and the predictive value, the most difficult prediction problem is CV0 (prediction all the lines in one environment, followed by CV1 (prediction of certain % of unobserved lines in all environments), and then CV2 (prediction some % of lines that were observed in some environments but not observed in other environments). When comparing prediction accuracies obtained by implementing different models the E + L model had the lowest accuracy for most of the traits when the CV1 and CV2 schemes were implemented, except trait SY, for which the implementation of CV1 with model E + L + G1 + LE produced the lowest prediction accuracy (0.087, Table 1), whereas the model L + E gave a prediction accuracy of 0.093 (Table 1).

**Table 1:** Mean prediction accuracy across 9 environments (site-by-year-by-management combination) for 13 models, 8 traits and 3 different cross-validation schemes (CV1, CV2 and CV0) for a chickpea population of 320 lines.

CV scheme	Traits	E + L		E + L + G1		E + L + G2		E + L + G3		E + L + G1 + LE		E + L + G2 + LE		E + L + G3 + LE		E + L + G1 + G1E		E + L + G2 + G2E		E + L + G3 + G3E		E + L + G1 + G1E + LE		E + L + G2 + G2E + LE		E + L + G3 + G3E + LE	
		Mean	SD	Mean	SD	Mean	SD	Mean	SD	Mean	SD	Mean	SD	Mean	SD	Mean	SD	Mean	SD	Mean	SD	Mean	SD	Mean	SD	Mean	SD
CV0	PH	0.355	—	0.363	—	0.376	—	0.366	—	0.363	—	0.378	—	0.367	—	0.357	—	0.364	—	0.354	—	0.359	—	0.376	—	0.357	—
	BM	0.170	—	0.192	—	0.204	—	0.196	—	0.193	—	0.207	—	0.199	—	0.161	—	0.183	—	0.166	—	0.171	—	0.165	—	0.163	—
	DF	0.458	—	0.475	—	0.476	—	0.477	—	0.476	—	0.474	—	0.476	—	0.442	—	0.463	—	0.447	—	0.455	—	0.437	—	0.442	—
	DM	0.247	—	0.250	—	0.248	—	0.249	—	0.252	—	0.245	—	0.247	—	0.244	—	0.215	—	0.243	—	0.249	—	0.253	—	0.249	—
	HI	0.078	—	0.080	—	0.068	—	0.077	—	0.077	—	0.062	—	0.071	—	0.075	—	0.078	—	0.083	—	0.081	—	0.066	—	0.078	—
	PS	0.096	—	0.132	—	0.144	—	0.135	—	0.136	—	0.151	—	0.141	—	0.108	—	0.127	—	0.105	—	0.109	—	0.122	—	0.115	—
	100—SDW	0.623	—	0.630	—	0.633	—	0.632	—	0.630	—	0.632	—	0.631	—	0.624	—	0.624	—	0.618	—	0.619	—	0.629	—	0.620	—
	SY	0.093	—	0.106	—	0.128	—	0.110	—	0.106	—	0.130	—	0.112	—	0.082	—	0.106	—	0.087	—	0.092	—	0.107	—	0.093	—
CV1	PH	−0.060	0.054	0.262	0.018	0.325	0.015	0.282	0.018	0.258	0.020	0.323	0.015	0.278	0.018	0.345	0.023	0.388	0.017	0.379	0.022	0.344	0.025	0.389	0.018	0.380	0.019
	BM	−0.066	0.061	0.178	0.019	0.206	0.013	0.191	0.017	0.174	0.021	0.204	0.014	0.187	0.020	0.230	0.027	0.260	0.026	0.251	0.027	0.226	0.028	0.257	0.025	0.244	0.030
	DF	−0.055	0.052	0.375	0.013	0.399	0.010	0.394	0.010	0.374	0.013	0.400	0.010	0.394	0.011	0.443	0.019	0.454	0.018	0.476	0.017	0.440	0.020	0.450	0.020	0.473	0.021
	DM	−0.039	0.059	0.168	0.028	0.155	0.023	0.171	0.026	0.162	0.028	0.153	0.026	0.166	0.031	0.282	0.024	0.331	0.022	0.315	0.022	0.276	0.025	0.330	0.022	0.309	0.024
	HI	−0.092	0.057	−0.023	0.055	0.037	0.032	−0.008	0.049	−0.027	0.055	0.034	0.038	−0.009	0.056	0.079	0.033	0.146	0.028	0.110	0.033	0.078	0.038	0.143	0.029	0.109	0.032
	PS	−0.102	0.056	0.135	0.038	0.155	0.024	0.144	0.033	0.127	0.037	0.151	0.027	0.137	0.036	0.127	0.037	0.151	0.027	0.137	0.036	0.148	0.136	0.159	0.029	0.148	0.033
	100−SDW	−0.054	0.068	0.554	0.005	0.577	0.006	0.570	0.004	0.553	0.005	0.577	0.006	0.570	0.004	0.652	0.010	0.632	0.015	0.671	0.013	0.652	0.011	0.633	0.015	0.670	0.012
	SY	0.093	0.032	0.144	0.020	0.144	0.020	0.113	0.029	0.087	0.036	0.141	0.023	0.107	0.036	0.200	0.024	0.224	0.025	0.220	0.023	0.195	0.026	0.220	0.025	0.216	0.025
CV2	PH	0.328	0.017	0.347	0.015	0.369	0.013	0.351	0.014	0.348	0.016	0.370	0.012	0.352	0.014	0.412	0.017	0.434	0.016	0.432	0.019	0.412	0.015	0.435	0.016	0.430	0.021
	BM	0.139	0.028	0.174	0.025	0.196	0.021	0.181	0.023	0.176	0.024	0.198	0.022	0.183	0.024	0.221	0.020	0.245	0.020	0.237	0.021	0.221	0.021	0.247	0.022	0.236	0.022
	DF	0.422	0.020	0.461	0.016	0.472	0.013	0.467	0.015	0.462	0.016	0.472	0.012	0.468	0.014	0.533	0.015	0.537	0.016	0.551	0.014	0.533	0.016	0.539	0.016	0.552	0.015
	DM	0.229	0.020	0.237	0.020	0.239	0.020	0.238	0.020	0.238	0.019	0.240	0.020	0.239	0.021	0.336	0.021	0.391	0.027	0.362	0.019	0.336	0.020	0.394	0.021	0.361	0.020
	HI	0.053	0.027	0.063	0.027	0.074	0.026	0.064	0.028	0.059	0.028	0.074	0.027	0.063	0.030	0.114	0.028	0.172	0.030	0.139	0.029	0.113	0.029	0.170	0.031	0.137	0.030
	PS	0.068	0.030	0.114	0.027	0.135	0.024	0.120	0.026	0.114	0.029	0.136	0.025	0.120	0.026	0.130	0.032	0.151	0.029	0.138	0.031	0.130	0.032	0.149	0.028	0.137	0.032
	100−SDW	0.605	0.012	0.622	0.009	0.632	0.007	0.626	0.008	0.622	0.009	0.632	0.007	0.626	0.008	0.759	0.007	0.753	0.010	0.773	0.007	0.759	0.007	0.754	0.011	0.773	0.008
	SY	0.063	0.026	0.092	0.024	0.123	0.022	0.099	0.023	0.093	0.025	0.126	0.023	0.101	0.025	0.181	0.025	0.204	0.027	0.200	0.026	0.181	0.027	0.205	0.027	0.201	0.026

100−SDW- 100 Seed Weight; BM- Biomass; DF- Days to 50% Flowering; DM- Days to Maturity; HI- Harvest Index; PH- Plant Height; PS- number of Plant Stand; and SY- Seed Yield.

In general, the CV0 cross validation scheme produced highly variable prediction accuracy of models including or not the G × E interaction. For example, the best predictive models were found for DF (correlation of 0.477) and 100-SDW (correlations of 0.633) traits when predicting, on average, one environment with the other 8 environments comprises the main effects of E, L, G3 and G2. For traits PH and BM, a relatively low correlation was found for model E + L + G2 + LE (0.378, and 0.207, respectively,although model E + L + G2 + G1E + LE had a correlation of 0.376 for PH. For trait DM, the best predictive model was the interaction model E + L + G2E + LE (0.253) closely followed by model E + L + G1 + LE (0.252). Predicting one entire environments using the other 8 environments in the training set provided low prediction accuracies for traits HI, PS and SY. In terms of marker systems, no clear patterns could be identified based on CV0 prediction accuracy.

Results of random cross-validation CV1 indicated a more clear pattern in terms of model prediction accuracy (including G × E interaction) and marker systems (G2). For traits PH and BM, the best two predictive models were E + L + G2 + G2E and E + L + G2 + G2E + LE that had prediction accuracies 0.388 and 0.389, respectively for PH and 0.260 and 0.257, respectively for BM. Similar prediction accuracies for models and marker systems were found for traits DM, HI, and SY. On the other hand, for trait 100-SDW, the best predictive model and marker system was E + L + G3 + G3E + LE (0.670). Results from random cross-validation CV2 indicated that model E + L + G2 + G2E + LE gave high prediction accuracy to traits PH, BM, DM, HI, PS, and SY and model E + L + G3 + G3E + LE was the best model for traits DF and 100-SDW. For traits HI and PS, the model E + L + G2 + G2E showed the highest performance 0.172 and 0.151, respectively.

In the case of PH, the naïve and informed interaction model produced the highest prediction accuracies with both the CV1 and CV2 schemes. For PS the informed interaction model produced the highest prediction accuracy with the CV1 scheme, and the naïve interaction model produced the highest prediction accuracy when CV2 was implemented. All the traits produced the lowest prediction accuracies while implementing CV1 and CV2 scheme with main effect model except SY which did not produce the lowest prediction accuracy with CV1. Whereas implementation of CV0 scheme showed the contrasting observation of lowest prediction accuracies for all the traits except PS. Only PS produced the lowest prediction accuracies when implementing the CV0 scheme with main effect model, whereas the rest of the traits showed lower prediction accuracies with other GS models. For instance, the main effect model extended with naïve interaction (E + L + G + LE) produced the lowest prediction accuracies for HI with the CV0 scheme. Similarly for PH, BM, DM, 100-SDW and SY, the main effect model extended with informed interaction produced the lowest prediction accuracies, and the main effect model extended with the naïve and informed interaction produced the lowest prediction accuracies for DF.

While comparing CV0, CV1, and CV2 for the different traits and different GS models, it was observed that for five traits (PH, DF, DM, HI, 100-SDW) the maximum prediction accuracy always occurred when CV2 was used and for the remaining three traits (BM, PS, SY) it occurred when CV1 was used, and in all cases either models E + L + G + GE or E + L + G + GE + LE accounted for the maximum prediction accuracies. CV1 always resulted in prediction accuracy close to zero for some models as well as traits, but CV0 and CV2 did not. The main effect model across all the three CV schemes, for all the traits except SY, accounted for lowest prediction accuracies with the CV1 scheme.

### Comparison of genotyping platforms on prediction accuracies

Application of different genotyping platform *viz*. GBS (G1), DArTseq (G2) and GBS together with DArTseq (G3) had a clear impact on the prediction accuracies. The DArTseq was found performing consistently better than the GBS platform among the main effect models E + L + G, E + L + G + LE, E + L + G + GE and E + L + G + LE + GE when CV1 and CV2 were implemented, whereas certain models for certain traits performed best with G1 in comparison to G2 and G3 when the CV0 scheme was implemented. For instance for BM and DF, G1 produced the best prediction accuracies in E + L + G + LE + GE in comparison to G2 and G3. GBS produced the lowest prediction accuracies among most of the interaction models. It was either DArTseq (in most of the cases) or GBS combined with DArTseq accounting for highest prediction accuracies (Table 1).

### Impact of environment on prediction accuracies

On comparing the impact of different environments (year by location combinations) on prediction accuracies, trends were consistent among the different cross validation schemes, except for model E + L which lacks prediction accuracy under the CV1 scheme. Even though the models perform differently across environments, for most of the traits certain environments were identified that consistently resulted in the highest prediction accuracy. The prediction accuracies varied depending on the different cross validation schemes. For instance, the highest prediction accuracies were obtained for DF in environment ICRISAT12 regardless of CV scheme used (with an exception of the E + L model when the CV1 scheme was used). Similarly in the case of SY, the predictions were best for environment IARI12.

When the CV0 scheme was implemented it was hard to identify a superior model for the eight traits. For instance, on the implementation of CV0 model E + L + G2 + LE produced the highest prediction accuracies for four traits (PH, BM, PS, and SY) but the difference between the prediction accuracy for models were not significant. However, for all of the traits other than DF the model that resulted in the highest prediction accuracy included the G2 term.

For CV1, the model E + L + G2 + G2E had the highest prediction accuracy for four traits (BM, DM, HI and SY), the model L + E + G2 + G2E + LE had the highest prediction accuracy for two traits (PH and PS), and the model L + E + G3 + G3E had the highest prediction accuracy for the remaining two traits (DF and 100SDW).

For CV2, the model E + L + G2 + G2E + LE produced the highest prediction accuracy for four traits (PH, BM, DM, and SY). For most traits using the DArTseq data resulted higher prediction accuracies than using GBS data or the combination of GBS and DArTseq data.

**Figure 1:**
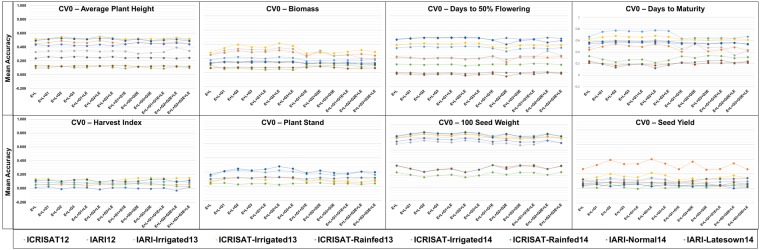
Prediction accuracy in a trial basis (within environment) of a chickpea population comprising 320 genotypes tested in 9 environments for nine models and eight traits under CV0 scheme (prediction of unobserved/new environments).

**Figure 2:**
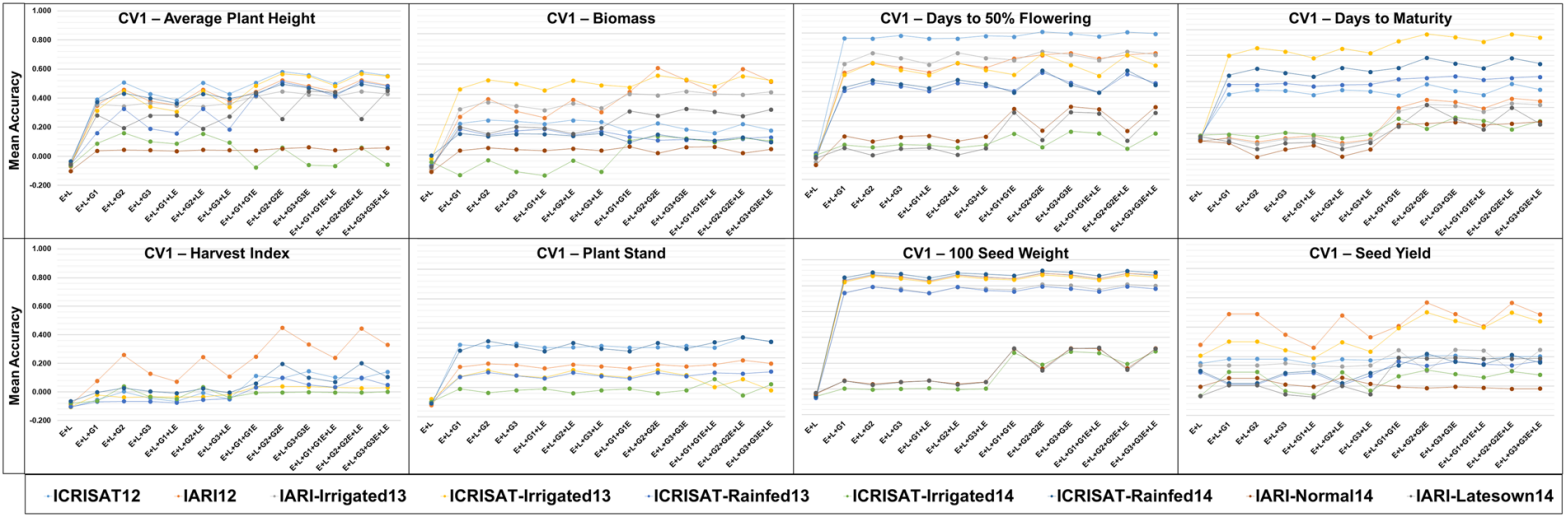
Prediction accuracy in a trial basis (within environment) of a chickpea population comprising 320 genotypes tested in 9 environments for nine models and eight traits under CV1 scheme (prediction of unobserved/new genotypes).

**Figure 3:**
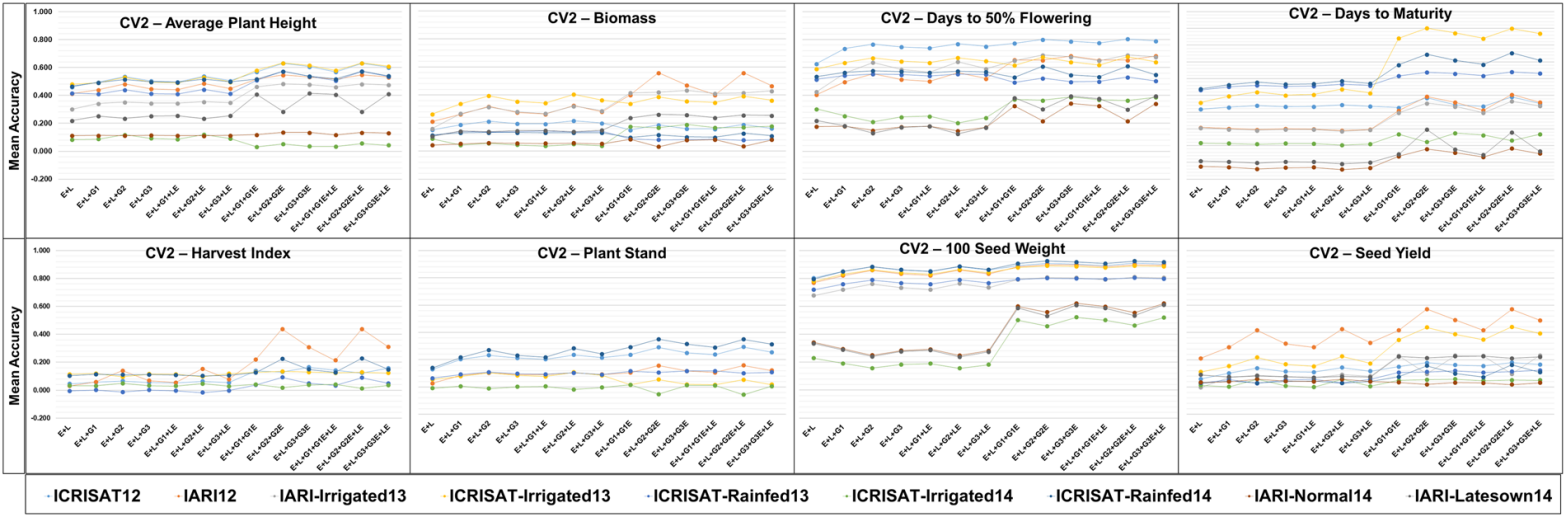
Prediction accuracy in a trial basis (within environment) of a chickpea population comprising 320 genotypes tested in 9 environments for nine models and eight traits under CV2 scheme (incomplete field trials - prediction of observed genotypes in observed environments).

The mean accuracy for the eight traits on the implementation of three CV schemes *viz*. CV0, CV1, and CV2 varied significantly (Figs 1–3). Within each panel variation in terms of predictions accuracies among the models and environments can be observed. There was a higher variation in terms of the mean prediction accuracy among the methods and environments when CV1 and CV2 were implemented compared to prediction accuracy for the CV0 scheme, and the variation is the highest for CV1. For SY, we could notice that when the CV0 scheme was utilized there was no significant difference between the schemes, and except to environment IARI12 all environments performed similarly in terms of prediction accuracy. The mean accuracy varied between 0 and 0.2 for most of the environments. Adding extra terms to the model did not improve the accuracy when CV0 was implemented. On implementation of the CV1 scheme a significant improvement in term of prediction accuracy could be observed on inclusion of the marker information and the interaction terms, compared to the simple main effect (E + L) model. Environment IARI12 performed the best in terms of prediction accuracies for SY. However, there was not a significant overall improvement in terms of prediction accuracy for trait SY in most of the environments for CV1 when we compared to CV0.

The mean accuracy of prediction improved for CV2 for the last six models i.e models including informed interactions, and informed and naïve interactions. For most of the environments the mean accuracy was between 0 and 0.2, and environment IARI12 performed the best in terms of prediction accuracy. A significant difference was observed among CV0, CV1, and CV2 for SY; CV1 and CV2 had a significantly higher prediction accuracy than CV0 for models with interaction terms GE and LE, and there was not a significant difference among the models using DArTseq versus GBS data.

While comparing all of the other traits, no significant increase was observed for any model, but the prediction accuracy was higher for some environments (Fig. 1). However, we could not identify any specific environment that consistently showed the highest prediction accuracy across the traits. For traits DF and 100-SDW, environments IARI-Latesown14, ICRISAT-Irrigated14, and IARI-Normal14 showed lower mean accuracy values in comparison to the rest of the environments across all models with all the CV schemes (Fig. 2). Most traits showed a similar pattern to SY for CV1 when we compared the models, but for some environments the prediction accuracy improved significantly, and reached 0.9 (eg. 100-SDW in ICRISAT-Rain14). When CV2 was implemented (Fig. 3) we could see that for DF and 100-SDW the environments were clustered into two groups based on their prediction accuracies. Environments IARI-Late14, ICRISAT-Irrig14, and IARI-Norm14 performed better than all of the other environments for these two traits.

## Discussion

Conventional breeding coupled with genomic tools has evolved in modern breeding approaches, offering precise selection of genotype in endeavor to develop superior lines. Traditionally, breeding programs used to undertake line selection based on breeding values taking into account the pedigree and the heritability of the trait (considering only the phenotyping data)^31^. However, conventional methods have several pitfalls including costs, labour and efforts of accurate phenotyping, for handling complex traits. Advancement in NGS technology has significantly reduced the genotyping cost, resulting in the generation of large amount of genotyping data, and it has further drawn a wide interest of researchers towards livestock and plant breeding^32^. Availability of the genotyping data, especially information about genomic regions involved in governing traits, aid better precision in selection. Molecular breeding approaches like MAS, MABC, and marker assisted recurrent selection (MARS) have been successfully deployed in many crop plants including legumes for trait improvement^33^. However, these approaches are only successful for traits with simple genetic behavior whereas addressing complex traits that have an extensive amount of small and large effect QTLs with MABC and MARS remain problematic. GS is another modern breeding approach that performs selection using genome-wide marker data, and has the potential to address complex traits. GS allows prediction of performance of individuals utilizing genome wide marker data instead of utilizing a limited number of markers with large effect as used to be the case in traditional MAS approaches^15,20^.

GS has been proven to outperform phenotype based selections in terms of cost as well as for enhancing the rate of genetic gain. GS results in shortening of the length of the breeding cycle by predicting the breeding value without evaluating in field and therefore saving large amount of resources^34–36^. Accuracies of genotyping in comparison to phenotyping enhances the accuracy of predicted breeding values, hence make selection process accurate and more precise. However high quality phenotyping facilities, when integrated with advanced high throughput genotyping platforms holds the potential to enhance the prediction further. The large number of markers enhance the precision of GS, hence the population size^37^, marker types and number^38,39^, statistical models^21^ etc., are some of the critical factors that determine the success of GS experiments.

GS efforts are being initiated for enhancing the rate of genetic gain among livestock, various crops including legumes^16,17,35,40–43^. Advances in sequencing technology have revolutionized the chickpea genomics in such a way that a crop that used to fall in the orphan crop category in terms of marker availability, has now become a genome resource rich crop^33^. Large genome resources further make GS a better-suited molecular breeding approach for chickpea. In order to deploy the markers in chickpea breeding using GS approaches, efforts were made to standardize the GS models for yield and yield related traits using a set of 320 elite chickpea lines^10^. The present study targeted eight yield related traits having agronomic importance in terms of the estimation of the effect of different genotyping methods as well as the effect of the environment on prediction accuracy. Results from the current study validated the results from a previous study using DArTseq the occurrence of two major groups in dendrogram using GBS data. In the dendrogram, two major clusters were observed when GBS data were used. Similar occurrence of two major clusters was observed in our previous study where *silico*DArT and DArTseq data were used^10^. Based on our previous study outcome, desi and kabuli were considered as a single set to calculate the prediction accuracies.

GBS has been a cost- and time-effective genotyping method for generating high density genotyping data for crop plants. GBS offers significant advantage over other genotyping methods, and has been successfully used for high density genetic mapping^44^ in chickpea and crop improvement efforts for GS in other species^36,45^. However, due to the high rate of missing data, the applicability of GBS for crop improvement is restricted and is being used by imputing the missing data, which sometimes affect the prediction accuracies. DArT (Diversity Array Technology) has been very useful in delineating the genetic diversity in chickpea^46^, and it has been used for initiating the GS efforts in chickpea^10^. Three different genotyping configurations (GBS, DArTseq, and combined genotyping data from DArTseq and GBS) were used in the present study to estimate the prediction accuracy for thirteen different statistical models.

Multiple variables ranging from environmental component to genomic components have an impact on genetic gain of crop plant and GS offers an opportunity to consider multiple variables simultaneously resulting in enhanced prediction accuracies^47,48^. Thus, different types of genotyping platforms and selection models with different interaction components *viz*. naïve and informative interactions were assessed in the current study. Higher prediction accuracies were obtained with models where only DArTseq data (G2) were considered, in comparison to models considering GBS data and the combined GBS - DArTseq data for traits PH, BM, DM, PS and SY. The possible reason for this observation can be the occurrence of large amount of missing data in GBS. Whereas small deviations from the pattern were observed for trait 100-SDW with the model with the naïve and informed interactions resulted in higher prediction accuracies using G3 than using G2 for both the CV1 and CV2 schemes. Similarly for PS, when CV0 was implemented with the model including the naïve and informed interaction prediction accuracies based on G3 were higher than G2. In the case of HI, CV0 consistently produced higher prediction accuracies for G3 based models than G2 based models regardless of any kind of interaction, whereas for CV1 and CV2 the reverse pattern was observed. DM could not display any pattern in prediction accuracies.

Combining the advanced high throughput genotyping platforms for generating genotyping data, and the integration of it with multi-location phenotyping data can help in the process of dissecting the complex quantitative traits and further allow assessing the impact of interactions of genotype with changing climatic conditions. Inclusion of various environmental variable further strengthen the possibility to make GS model more accurate^49^ and further allow the possibility to predict the performance of the test population under environmental condition that have not been sampled for the genotype.

Three different cross validation schemes have been used in the current study based on the prediction problems that plant breeder often encounters *viz*. (1) performance of prediction of untested genotypes (CV1); (2) performance of prediction of genotypes tested in some but not in other environments (CV2); (3) performance of prediction of tested genotypes in new environments (CV0). Likewise previous studies, performance of prediction of untested genotypes (CV1) resulted in lower prediction accuracies in most of the traits with different GS models in comparison to the other two cross validation schemes. It could be due to absence of the information from correlated environments which was considered in the rest of the schemes. As suggested earlier selection of cross validation will further have an impact on rate of genetic gain. For instance CV1 may result in selection of new lines without field testing, but will also result in poor predictive value which may further affect the rate of genetic gain^29^.

Considering the findings of the current study, there is a need to deploy models that take into consideration the impact of different environmental conditions at multiple locations for multiple years. Thus the model considering G × E effects may further improve the prediction accuracies^50,51^. Inclusion of the G × E effect in GS models holds the true potential to enhance GS in practice.

## Methods

### Phenotypic data

**Figure 4:**
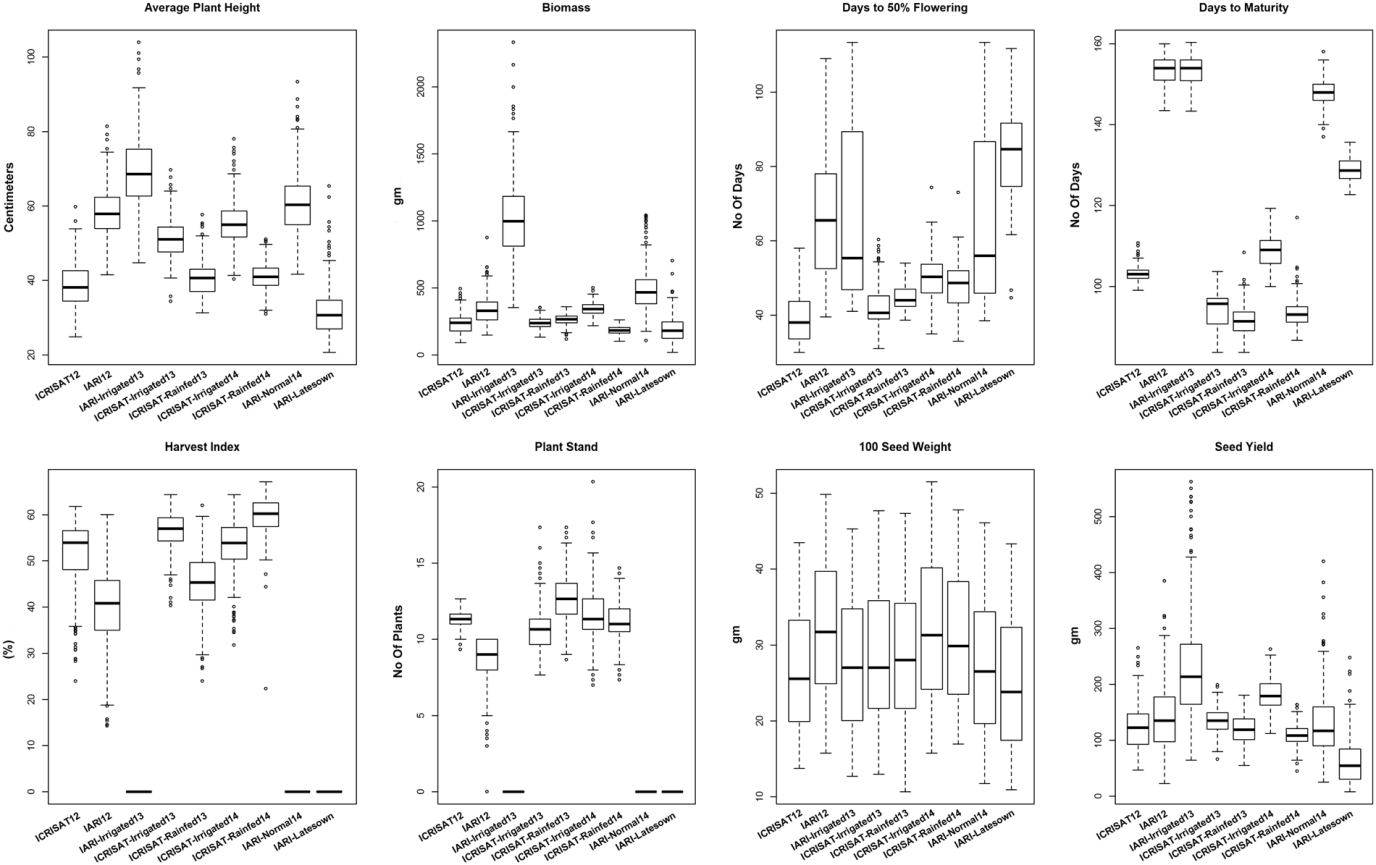
Graphical representation of phenotypic data on eight traits (100 Seed Weight (100-SDW), Biomass (BM), Days to 50% Flowering (DF), Days to Maturity (DM), Harvest Index (HI), Plant Height (PH), number of Plant Stand (PS), and Seed Yield (SY)) analyzed for three seasons at IARI, New Delhi and ICRISAT, Patancheru.

A set of 320 elite chickpea breeding lines including both desi and kabuli seed types from the International Chickpea Screening Nursery (ICSN) of ICRISAT were used in this study (as described in Roorkiwal *et al*.^10^). These lines were extensively phenotyped for three seasons (2012-13, 2013-14 and 2014-15) at two different geographical locations namely ICRISAT, Patancheru (17°31′48.00″N 78°16′12.00″E) and IARI, New Delhi (28.6374°N, 77.1629°E) in India. Phenotypic data on eight traits (100 Seed Weight (100-SDW), Biomass (BM), Days to 50% Flowering (DF), Days to Maturity (DM), Harvest Index (HI), Plant Height (PH), number of Plant Stand (PS), and Seed Yield (SY)), on these 320 lines under different water regimes (normal-rainfed, irrigated and late sown) were used for analysis (Fig. 4). Environments were defined as the location-by-year-by-water management combination, and 9 different combinations were observed (Table 2).

**Table 2:** Trials/environments as result of year-by-location/management combination.

Year	Management	Location		Environment	
		IARI	ICRISAT	IARI	ICRISAT
2012	Normal	X	X	IARI12	ICRISAT12
2013	Irrigated	X	X	IARI-Irrig13	ICRISAT-Irrig13
	Rainfed	X			ICRISAT-Rain13
2014	Irrigated	X			ICRISAT-Irrig14
	Rainfed	X			ICRISAT-Rain14
	Normal		X	IARI-Norm14	
	Latesown		X	IARI-Late14	

### Genotyping and SNP calling

High quality genomic DNA was isolated from the plant leaves collected from 15 days old seedlings using high throughput mini-DNA extraction method^52^. Quality and quantity of DNA were assessed using spectrophotometer (Shimadzu UV160A, Japan). All 320 lines with high-quality DNA were selected for sequencing using the GBS approach as described by Elshire *et al*.^53^. The GBS libraries for all 320 lines were prepared by digesting genomic DNA with *ApeKI* endonuclease (recognition site: G/CWCG). T4 DNA ligase was used for ligating uniquely barcoded adaptors with digested DNA fragments. Equal proportion of barcoded adaptors ligated DNA fragments from each sample were mixed for GBS libraries construction, which were amplified, purified in order to remove excess adapters, followed by sequencing on the HiSeq 2500 platform (Illumina Inc, San Diego, CA, USA). The reads obtained were analyzed using the TASSEL-GBS pipeline implemented in TASSEL 4.0^54^. Sequence reads were first de-multiplexed based on the sampled barcodes and trimmed to the first 64 bases starting from enzyme cutting site, using in-house perl scripts. Sequence reads with presence of ‘N’ within the first 64 bases were not taken into consideration. Reads with more than 50% of low quality base pairs (Phred <5%) were discarded, and filtered data were used for SNP calling. The remaining good quality reads (called tags) were aligned against draft genome sequence (CaGAv1.0) of chickpea^1^ using the Bowtie 2 software^55^. Using GBS analysis pipeline alignment file was processed for SNP calling and genotyping. An allele was considered only if it was supported with a minimum tag count value of 10. The SNPs identified were further filtered to remove missing data and such filtered SNPs were used for further application.

In addition to GBS, DArTseq data on 320 lines described by Roorkiwal *et al*.^10^ were also used for analysis. In summary; data from two different platforms were used for analysis: (1) GBS data for 88 K SNPs denoted by G1, (2) DArTseq with 1.6 K SNPs denoted by G2, and (3) GBS data combined with DArTseq data denoted by G3.

### Statistical Models

Variants of the MRNM by Jarquín *et al*.^30^ were used for predictions. A total of thirteen models were fit; four of these models included only main effects, three included naïve interactions between genotype and environments (with no marker data involved in the interaction component), and the remaining six models included marker information in the interactions. The genomic models used the genomic matrix based on either the GBS or DArTseq data, or both the DArTseq and GBS to establish the relationships among pairs of genotypes and allow borrowing information among lines. Conceptually, the models can be described as follows: a basic model (E + L) which included the main effects of environments (E) and lines (L); a model (E + L + G) also including the main effects of markers (G); a naïve (genotype by environment) interaction model (E + L + G + LE), and an informed (marker by environment) interaction model (E + L + G + LE + GE). As described before, only the type of platform (GBS or DArTseq or both) were varied for the models that included the genomic component. Further details for all of the models are given below.

### Main effects models

#### Main effects of environments and lines (E + L)

The response of the phenotypes (*y*_*ij*_) defined by random baseline model is

1 $${y}_{ij}=\mu +{E}_{i}+{L}_{j}+{e}_{ij}$$

where *μ* is the overall mean, *E*_*i*_ is the random effect of the *i*^th^ environment, *L*_*j*_ is the random effect of the *j*^th^ line, *EL*_*ij*_ is the interaction between the *i*^th^ environment and the *j*^th^ line, and *e*_*ij*_ is the random error term. All random effects follow an independent and identically distributed (iid) multivariate normal distribution such that ${E}_{i} \sim N(0,{\boldsymbol{I}}{\sigma }_{E}^{2})$, ${L}_{j} \sim N(0,{\boldsymbol{I}}{\sigma }_{L}^{2})$, and ${e}_{ij} \sim N(0,{\boldsymbol{I}}{\sigma }_{e}^{2})$ where ${\sigma }_{E}^{2}$,$\,{\sigma }_{L}^{2}$, ${\sigma }_{e}^{2}\,$are the environment, line, and residual variances, respectively. The baseline model (1) could have included the line × environments interaction $\,E{L}_{ij} \sim N(0,{\boldsymbol{I}}{\sigma }_{EL}^{2})$, where ${\sigma }_{EL}^{2}$ is the line × environment interaction variance.

In the model above, the random effect of the line (*L*_*j*_) can be replaced by *g*_*j*_, which is an approximation of the genetic value of the *j*^*th*^ line from the genomic relationship matrix [also, the effects of the line (*L*_*j*_) can be replaced by *a*_*j*_, which is the additive effect obtained from the pedigree information]. In the models described below, we can use *g*_*j*_ as well as its interactions with environment $\,{E}_{i}(g{E}_{ij})$. Full descriptions of the different reaction norm models can be found in Jarquin *et al*.^30^. Below we give a brief description of the different models that were fitted using genomic information.

#### Models including the main effects of GBS (E + L + G1), DArTseq markers (E + L + G2) and both DArTseq markers and GBS SNPs (E + L + G3)

These models were fitted by adding the genomic random effect of the line *g*_*j*_ to the previous model described by equation (1). This was an approximation of the genetic value of the *j*^*th*^ line, and is defined by the regression of marker covariates ${g}_{j}=\sum _{m=1}^{p}{x}_{jm}{b}_{m}$, where *x*_*jm*_ is the genotype of the *j*^*th*^ line at the *m*^*th*^ marker position (either from G1, G2 or G3), and *b*_*m*_ is the effect of the *m*^*th*^ marker assuming that ${b}_{m}\mathop{ \sim }\limits^{IID}N(0,{\sigma }_{b}^{2})$ (*m* *=* 1, …, *p)*, with ${\sigma }_{b}^{2}$ being the common variance of the marker effects. The vector *g* = (*g*_1_, …, *g*_*j*_)′ contains the genomic values of all the lines and by properties of the multivariate normal distribution it follows a multivariate normal density with zero mean and covariance matrix $Cov({\boldsymbol{g}})={\boldsymbol{G}}{\sigma }_{g}^{2}$, where ***G*** is the genomic relationship matrix, and ${\sigma }_{g}^{2}\propto {\sigma }_{b}^{2}$ is proportional to the genomic variance. The model with the environmental effect, line effect, and genomic effect could be written as

2 $${y}_{ij}=\mu +{E}_{i}+{L}_{j}+{g}_{j}+{e}_{ij}$$

where *g*_*j*_ is a random variable that allows borrowing information between lines through genomic information. Specifically, vector **g** = (*g*_1_, …, *g*_*j*_)′ has the genomic value of the lines and it is assumed to follow a multivariate normal distribution such that ${\boldsymbol{g}} \sim N(0,{\boldsymbol{G}}{\sigma }_{g}^{2})\,\,$where ${\sigma }_{g}^{2}\,$is the genetic variance of the lines and **G** = $\frac{{\boldsymbol{X}}{{\boldsymbol{X}}}^{{\boldsymbol{^{\prime} }}}}{p}$, with ***X*** as the centered and standardized matrix of molecular markers where *p* is the number of markers. The parameterization of this component is also known as the Genomic Best Linear Unbiased Predictor (GBLUP) model^56,57^. The random effects ***g*** = (*g*_1_, …, *g*_*j*_)′ are correlated such that model (2) allows exchanging information across lines.

Note that the term *g*_*j*_ should account for the additive genetic effects and it approximates the true genetic values of the *L*_*j*_ line; the main effect of the lines also include non-additive effects that are not accounted by *g*_*j*_ obtained from the linear kernel GBLUP. When the phenotype being model is controlled by additive genetic effects, *L*_*j*_, can be dropped from the model. Here we choose to keep *L*_*j*_, to account for any non-additive genetic effects influencing the phenotypes being modeled.

### Main effects and interaction models

#### Models E + L + G1, E + L + G2 and E + L + G3 with extended naïve interactions (E + L + G1 + LE), (E + L + G2 + LE) and (E + L + G3 + LE)

Models E + L + G1 + LE, E + L + G2 + LE and E + L + G3 + LE are similar to models E + L + G1, E + L + G2 and E + L + G3 respectively but include the interaction of the *j*^th^ line and the *i*^th^ environment *EL*_*ij*_. The model with interaction can be written as an extension of model (2)

3 $${y}_{ij}=\mu +{E}_{i}+{L}_{j}+{g}_{j}+E{L}_{ij}+{e}_{ij}$$

where the term *EL*_*ij*_ denotes the interaction of the *j*^th^ line and the *i*^th^ environment and the other terms are previously defined. The interaction term is assumed to have a normal distribution such that ${\boldsymbol{EL}} \sim N(0,({{\boldsymbol{Z}}}_{{\boldsymbol{L}}}{\boldsymbol{I}}{{\boldsymbol{Z}}}_{{\boldsymbol{L}}}^{{\boldsymbol{^{\prime} }}})^\circ ({{\boldsymbol{Z}}}_{{\boldsymbol{E}}}{{\boldsymbol{Z}}}_{{\boldsymbol{E}}}^{{\boldsymbol{^{\prime} }}}){\sigma }_{EL}^{2})$, where ***Z***_L_ and ***Z***_E_ are the incidence matrices for lines and environments, respectively, ${\sigma }_{EL}^{2}$ is the variance component of the interaction term *EL*, and ° denotes the Hadamar or Schur product (element by element product) between two matrices.

#### Models E + L + G1, E + L + G2 and E + L + G3 with informed interaction (between markers and environments (E + L + G1 + G1E), (E + L + G2 + G2E) and (E + L + G3 + G3E)

Models E + L + G1 + G1E, E + L + G2 + G2E and E + L + G3 + G3E were extended models of E + L + G1, E + L + G2 and E + L + G3 respectively. In models E + L + G1 + G1E, E + L + G2 + G2E and E + L + G3 + G3E a random interaction term is added between the random effect of the *i*^th^ environment (*E*_*i*_ and the *j*^th^ genomic component (*g*) of the lines denoted by *Eg*_*ij*_. The model can be written as

4 $${y}_{ij}=\mu +{E}_{i}+{L}_{j}+{g}_{j}+E{g}_{ij}+{e}_{ij}$$

where ${\boldsymbol{Eg}} \sim N(0,({{\boldsymbol{Z}}}_{{\boldsymbol{g}}}{\boldsymbol{G}}{{\boldsymbol{Z}}}_{{\boldsymbol{g}}}^{{\boldsymbol{^{\prime} }}})^\circ ({{\boldsymbol{Z}}}_{{\boldsymbol{E}}}{{\boldsymbol{Z}}}_{{\boldsymbol{E}}}^{{\boldsymbol{^{\prime} }}}){\sigma }_{Eg}^{2})$ conceptually represents the interaction between each genomic marker and each environment, ***Z***_*g*_ is the incidence matrix for the effects of the genomic values ***g***, and ${\sigma }_{Eg}^{2}$ is the variance component of ***Eg***. Matrix *Z*_*E*_ is the incidence matrix for the environments. As previously indicated the genomic matrix ***G*** is used to account for the genomic main effects and for the genotype × environment interaction effect, which could be either derived from marker systems G1, G2 or G3.

#### Models E + L + G1, E + L + G2 and E + L + G3 with naïve interaction and informed interaction (E + L + G1 + G1E + LE), (E + L + G2 + G2E + LE) and (E + L + G3 + G3E + LE)

Models E + L + G1 + G1E + LE, E + L + G2 + G2E + LE and E + L + G3 + G3E + LE are extensions of models E + L + G1, E + L + G2 and E + L + G3, respectively, and they include the interaction between the environments and lines denoted by *EL*_*ij*_, and the interaction between environments and the genomic values denoted by *Eg*_*ij*_. The model including the two interaction terms can be written as

5 $${y}_{ij}=\mu +{E}_{i}+{g}_{j}+{L}_{j}+E{L}_{ij}+E{g}_{ij}+{e}_{ij}$$

where all terms have been defined previously. In this model *Eg*_*ij*_ approximates the effect of *EL*_*ij*_, and its approximations will depend, among other factors, on the degree of linkage disequilibrium between the markers (or haplotypes) and the QTLs of the traits under study as well as the density and distribution of the markers or/and haplotypes in the genome.

### Prediction assessment by cross-validation

Three different random CV schemes were used in the present study. The first cross-validation (CV1) evaluates the prediction accuracy of models when a set of lines have not been assessed in any of the environments (prediction of newly developed lines)^29^. The second cross-validation scheme (CV2) evaluates the prediction accuracy of models when some lines have been evaluated in some of the environments but not in other environments (sparse testing). For the CV2 scheme, information from related lines and correlated environments is used, and the prediction assessment benefits from borrowing information from lines within an environment, from lines across environments, and from correlated environments^29^. The third cross-validation (CV0) scheme predicts an unobserved environment using the remaining environments as a training set (predict untested environments by leave-one-ouy system). Predictability is measured using the Pearson correlation coefficient between the observed phenotype and the predicted genomic breeding value.

In both CV1 and CV2, a five-fold CV scheme was used to generate the training and testing sets, and the prediction accuracy was assessed for each testing set. For the CV1 approach, lines were divided into five folds such that approximately 20% of lines were in one group so phenotypes from the same line appear in the same group thus when a genotype is not observed in all environments it is hard to have groups with the same sample size.

For CV2, phenotypes were randomly divided into five subsets, where 80% of the lines were assigned to the training set, and 20% were assigned to the testing set. Four subsets were combined to form the training set, and the remaining subset was used as the validation set. The permutation of the five subsets led to five possible training and validation data sets. This procedure was repeated 20 times, and a total of 100 runs were performed for each trait-environment combination on each population. The same partition was used for analysis with all the GS models. Prediction accuracy was assessed as the average value of the correlations between the phenotype and the GEBVs from 100 runs calculated in each population for each trait-environment combination.

For CV0, simulating the scenario of prediction of unobserved set of environmental conditions, the leave-one-environment out strategy was adopted. Here, each environment was predicted using the remaining environments. Since no random process is involved assigning folds the correlation between predicted and observed values within each environment was computed only once.

### Computational tools for analysis

The Bayesian Generalized Linear Regression (BGLR) R-package^22,58,59^ was used for fitting the described GS models. The package handles pedigree data in parametric and semiparametric contexts, thus allowing different random matrices with user defined covariance matrices. The scripts used are similar to those provided in Pérez-Rodríguez *et al*.^59^.

## References

[1] Varshney, R. K. *et al*. Draft genome sequence of chickpea (*Cicer arietinum* L.) provides a resource for trait improvement. *Nat. Biotechnol.* **31**, 240–246 (2013). 10.1038/nbt.2491 23354103

[2] Khatoon N, Prakash J (2004). Nutritional quality of microwave-cooked and pressure-cooked legumes. Int. J. Food Sci. Nutr..

[3] Jukanti, A. K., Gaur, P. M., Gowda, C. L. & Chibbar, R. N. Nutritional quality and health benefits of chickpea (*Cicer arietinum* L.): a review. *Br. J. Nutr.* **108**, S11–26 (2012). 10.1017/S0007114512000797 22916806

[4] Croser JS, Ahmad F, Clarke HJ, Siddique KHM (2003). Utilisation of wild Cicer in chickpea improvement - progress, constraints, and prospects. Crop Pasture Sci..

[5] Singh U (1985). Nutritional quality of chickpea (Cicer arietinum L.): current status and future research needs. Plant Foods Human Nut..

[6] Singh KB (1997). Chickpea (Cicer arietinum L.). Field Crops Res..

[7] Varshney RK, Graner A, Sorrells ME (2005). Genomics-assisted breeding for crop improvement. Trends Plant Sci..

[8] Thudi M (2016). Recent breeding programs enhanced genetic diversity in both desi and kabuli varieties of chickpea (Cicer arietinum L.). Sci. Rep..

[9] Thudi M (2016). Whole genome re-sequencing reveals genome-wide variations among parental lines of 16 mapping populations in chickpea (Cicer arietinum L.). BMC Plant Biol..

[10] Roorkiwal M (2016). Genome-enabled prediction models for yield related traits in chickpea. Front Plant Sci..

[11] Varshney RK (2013). Fast-track introgression of “QTL-Hotspot” for root traits and other drought tolerance traits in JG 11, an elite and leading variety of chickpea. The Plant Genome.

[12] Varshney RK (2014). Marker-assisted backcrossing to introgress resistance to fusarium wilt race 1 and ascochyta blight in C 214, an elite cultivar of chickpea. The Plant Genome.

[13] Desta ZA, Ortiz R (2014). Genomic selection: genome-wide prediction in plant improvement. Trends Plant Sci..

[14] Bernardo R, Yu J (2007). Prospects for genome-wide selection for quantitative traits in maize. Crop Sci..

[15] Meuwissen THE, Hayes BJ, Goddard ME (2001). Prediction of total genetic value using genome wide dense marker maps. Genetics.

[16] de los Campos G, Gianola D, Rosa GJM (2009). Reproducing kernel Hilbert spaces regression: a general framework for genetic evaluation. J. Anim. Sci..

[17] de los Campos G (2009). Predicting quantitative traits with regression models for dense molecular markers and pedigree. Genetics.

[18] Crossa J (2010). Prediction of genetic values of quantitative traits in plant breeding using pedigree and molecular markers. Genetics.

[19] Crossa J (2017). Genomic selection in plant breeding: Methods, models, and perspectives. Trends Plant Sci..

[20] Jannink J-L, Lorenz AJ, Iwata H (2010). Genomic selection in plant breeding: from theory to practice. Brief. Funct. Genomics.

[21] Heslot N, Yang HP, Sorrells ME, Jannink JL (2012). Genomic selection in plant breeding: a comparison of models. Crop Sci..

[22] de los Campos G, Hickey JM, Pong-Wong R, Daetwyler HD, Calus MP (2013). Whole-genome regression and prediction methods applied to plant and animal breeding. Genetics.

[23] Howard R, Carriquiry AL, Beavis WD (2014). Parametric and nonparametric statistical methods for genomic selection of traits with additive and epistatic genetic architectures. G3 (Bethesda).

[24] Windhausen VS (2012). Effectiveness of genomic prediction of maize hybrid performance in different breeding populations and environments. G3 (Bethesda).

[25] Xu S, Zhu D, Zhang Q (2014). Predicting hybrid performance in rice using genomic best linear unbiased prediction. Proc. Nat. Acad. Sci..

[26] Zhao Y, Mette MF, Gowda M, Longin CF, Reif JC (2014). Bridging the gap between marker-assisted and genomic selection of heading time and plant height in hybrid wheat. Heredity.

[27] Mishra US, Sirothia P, Bhadoria US (2009). Effects of phosphorus nutrition on growth and yield of chickpea (Cicer arietinum L) under rain fed conditions. Int. J. Agri. Stat. Sci..

[28] Bampidis VA, Christodoulou V (2011). Chickpeas (Cicer arietinum L.) in animal nutrition: A review. Animal Feed Sci. Tech..

[29] Burgueño J (2012). de los Campos, G., Weigel, K. & Crossa, J. Genomic prediction of breeding values when modeling genotype × environment interaction using pedigree and dense molecular markers. Crop Sci..

[30] Jarquín D (2014). A reaction norm model for genomic selection using high-dimensional genomic and environmental data. Theor. Appl. Genet..

[31] Hayes BJ, Lewin HA, Goddard ME (2013). The future of livestock breeding: genomic selection for efficiency, reduced emissions intensity, and adaptation. Trends Genet..

[32] Pérez-Enciso M, Rincón JC, Legarra A (2015). Sequence-vs. chip-assisted genomic selection: accurate biological information is advised. Genet. Select. Evol..

[33] Varshney RK (2016). Exciting journey of 10 years from genomes to fields and markets: Some success stories of genomics-assisted breeding in chickpea, pigeonpea and groundnut. Plant Sci..

[34] Heffner EL, Sorrells ME, Jannink JL (2009). Genomic selection for crop improvement. Crop Sci..

[35] Heffner EL, Lorenz AJ, Jannink JL, Sorrells ME (2010). Plant breeding with genomic selection: gain per unit time and cost. Crop Sci..

[36] Isidro J (2015). Training set optimization under population structure in genomic selection. Theor. Appl. Genet..

[37] Daetwyler HD, Villanueva B, Woolliams JA (2008). Accuracy of predicting the genetic risk of disease using a genome-wide approach. PloS One.

[38] Chen X, Sullivan PF (2003). Single nucleotide polymorphism genotyping: biochemistry, protocol, cost and throughput. Pharmacogenomics J..

[39] Poland J, Rife TW (2012). Genotyping-by-sequencing for plant breeding and genetics. The Plant Genome.

[40] Hayes B, Goddard M (2010). Genome-wide association and genomic selection in animal breeding. Genome.

[41] Goddard ME, Hayes BJ, Meuwissen TH (2010). Genomic selection in livestock populations. Genet. Res..

[42] Gorjanc G, Hickey JM, Cleveland MA, Houston RD (2015). Potential of genotyping-by-sequencing for genomic selection in livestock populations. Genet. Select. Evol..

[43] Jain, A., Roorkiwal, M., Pandey, M. & Varshney, R. K. Current status and prospects of genomic selection in legumes. In: Genomic Selection for Crop Improvement, R. K. Varshney *et al*. (eds), Springer International Publishing (2017).

[44] Jaganathan D (2015). Genotyping-by-sequencing based intra-specific genetic map refines a “QTL-hotspot” region for drought tolerance in chickpea. Mol. Gen. Genomics.

[45] Huang YF, Poland JA, Wight CP, Jackson EW, Tinker NA (2014). Using genotyping-by-sequencing (GBS) for genomic discovery in cultivated oat. PLoS One.

[46] Thudi M (2011). Novel SSR markers from BAC-end sequences, DArT arrays and a comprehensive genetic map with 1,291 marker loci for chickpea (Cicer arietinum L.). PLoS One.

[47] Bassi FM, Bentley AR, Charmet G, Ortiz R, Crossa J (2016). Breeding schemes for the implementation of genomic selection in wheat (Triticum spp.). Plant Sci..

[48] Bhat JA (2016). Genomic selection in the era of next generation sequencing for complex traits in plant breeding. Front. Genet..

[49] Pierre CS (2016). Genomic prediction models for grain yield of spring bread wheat in diverse agro-ecological zones. Sci Rep..

[50] Jonas E, de Koning DJ (2013). Does genomic selection have a future in plant breeding?. Trends Biotechnol..

[51] Oakey H (2016). Genomic selection in multi-environment crop trials. G3 (Bethesda).

[52] Cuc LM (2008). Isolation and characterization of novel microsatellite markers and their application for diversity assessment in cultivated groundnut (Arachis hypogaea). BMC Plant Biol..

[53] Elshire RJ (2011). A robust, simple genotyping-by-sequencing (GBS) approach for high diversity species. PloS One.

[54] Bradbury PJ (2007). TASSEL: software for association mapping of complex traits in diverse samples. Bioinformatics.

[55] Langmead B, Salzberg SL (2012). Fast gapped-read alignment with Bowtie 2. Nat. Methods.

[56] VanRaden PM (2007). Genomic measures of relationship and inbreeding. Interbull Bull..

[57] VanRaden PM (2008). Efficient methods to compute genomic predictions. J. Dairy Sci..

[58] Pérez-Rodríguez P, de los Campos G (2014). Genome-wide regression & prediction with the BGLR statistical package. Genetics.

[59] Pérez-Rodríguez P (2015). A pedigree-based reaction norm model for prediction of cotton yield in multi-environment trials. Crop Sci..

